# Correspondence on Lovell et al.: response to Bornelöv et al.

**DOI:** 10.1186/s13059-017-1234-y

**Published:** 2017-06-14

**Authors:** Peter V. Lovell, Claudio V. Mello

**Affiliations:** Department of Behavioral Neuroscience, Oregon Health and Science University, Portland, OR 97017 USA

## Abstract

While the analysis of Bornelöv et al. is informative, they provide evidence for the existence of only 3% of the reported avian missing genes set, and thus do not significantly challenge our main findings that specific groups of syntenic protein-coding genes are missing in birds.

This is a response to the Correspondence article: https://www.dx.doi.org/10.1186/s13059-017-1231-1

Lovell et al. [1] previously reported that a set of 274 protein-coding genes could not be found in the genomes of approximately 60 avian species. As many of the 274 ‘avian missing genes’ belong to syntenic blocks that are conserved in other vertebrates, we hypothesized that their loss might have occurred in blocks, possibly due to chromosomal rearrangements. Recently, Hron et al. [2] (using the Chicken Sequence Read Archive (SRA)) and Warren et al. [3] (using Galgal5) have found evidence for the existence of a limited set of these ‘avian missing genes’ in chicken, which has helped to refine our initial analysis. On the basis of searches of chicken transcriptome databases, Bornelöv et al. [4] now report the discovery of a set of 137 genes previously thought to be missing in birds. However, a close analysis reveals that the genes for which they provide evidence comprise less than 3% of the 274 genes in the ‘avian missing gene set’. The majority of their newly found genes are part of the lower confidence set that were absent from chicken but present in other birds (Table S6 in Lovell et al. [1]), or are derived from unrelated studies [5]. Thus, while the Bornelöv et al. [4] study is informative, it does not significantly challenge the finding that *specific* groups of syntenic protein-coding genes are missing in birds.

In our 2014 study [1], we exhaustively searched the approximately 60 avian genomes then available for several hundred protein-coding genes that are present in humans and lizards, but that were not predicted in the zebra finch (Taegut1) or chicken (Galgal4) genomes. Extensive BLAT and BLAST searches were performed against avian cDNA, protein, trace archive, and assembled genome databases using orthologous sequences from human, lizard, and crocodile as queries, in an attempt to find traces of these missing genes in any avian species. That analysis concluded that there was no evidence for 274 protein-coding genes in any bird species (Tables 1 and S1 in Lovell et al. [1]). Interestingly, the majority of these avian missing genes occur in syntenic clusters that are conserved among non-avian vertebrate species, or are in close proximity to such clusters. We thus proposed that those avian missing genes may have been lost in syntenic blocks, possibly as a result of chromosomal rearrangements. We also reported a second lower-confidence set of 174 genes (Table S6A and B in Lovell et al. [1]) that we were unable to find in the chicken genome (Galgal4) or transcriptome databases. As these genes were present in other birds, it seemed likely that they might eventually be found in chicken as sequencing technology improved. Indeed, gene reconstructions from chicken SRA transcriptome databases [2], and/or National Center for Biotechnology Information (NCBI) Gnomon gene predictions in the latest chicken genome assembly ([3], Galgal5) have recently provided evidence for 240 genes that were previously missing in the chicken genome (Table S5 in Warren et al. [3]), and have shown that some of these genes are expressed in various tissues. Despite these findings, the majority of genes (85%) that were reported as missing in all birds (Tables 1 and S1 in Lovell et al. [1]) remained missing (see also discussion in [6]).

Bornelöv et al. [4] now claim to have additional evidence from searches of chicken transcriptome databases for the existence of 137 genes previously thought to be missing in birds, and conclude that this finding significantly challenges the Lovell et al. [1] finding that a specific set of genes organized in conserved syntenic blocks are missing in birds. Specifically, Bornelöv et al. presents a ‘high-confidence set’ of 85 genes (Table 1 in Bornelöv et al. [4]) reconstructed from chicken transcriptome databases (the remaining genes from their set of 137 are apparently from an ‘intermediate set’ for which the supporting evidence is not readily apparent). A careful count reveals there are actually 74 non-redundant entries (Table 1 in Bornelöv et al. [4], ‘Predicted absent in birds [1]; Found in our high confidence list’). When we compared this list of 74 genes to the avian missing gene set described in Lovell et al. [1], we found that Bornelöv et al. provided evidence in chicken for eight genes among the 274 genes missing in the approximately 60 avian genome and transcriptome databases available at the time. This includes two genes (*LPPR2* and *NPHS1*) that were directly part of the avian missing syntenic blocks (Table S1A in Lovell et al. [1]) and six genes in close proximity to such blocks (*FLT3LG*, *PLCB3*, *PRSS8*, *RCN3*, *TRMT1*, and *TSPAN31*; Table S1B in Lovell et al. [1]). Notably, these eight genes account for about 3% of the originally reported 274 missing genes.

Bornelöv et al. also present transcriptome evidence for an additional 27 genes (Table 1 in Bornelöv et al., ‘Predicted absent in birds [2]; Found in our high confidence list’, after removing redundant *NPHS1* and *PLCB3* entries [4]) previously reported as missing in a separate study of 48 avian genomes by Zhang et al. [5]. Importantly, this set of 27 genes is non-overlapping with the genes from the missing syntenic blocks reported in Lovell et al. [1]. Thus, while these findings help to clarify losses reported in the study of Zhang et al., they bear little relevance to the presence or absence of missing syntenic gene blocks in chicken or other bird genomes.

Bornelöv et al. [4] also report transcriptome evidence for an additional 50 high-confidence genes that are reportedly missing in chicken, but not in all birds (Table 1 in Bornelöv et al. [4]). After removing 11 entries that are redundant with those listed in ‘Predicted absent in birds [2]; Found in our high confidence list’, we conclude that the authors were referring here to 39 genes previously not found in chicken (Galgal4), and thus not included among the genes reported as missing in syntenic blocks in all birds (Tables 1 and S1 in Lovell et al. [1]). Instead, Lovell et al. [1] reported these genes in a Supplemental Table (Table S6 in [1]) as a means of facilitating future investigations into possible gene losses in galliformes. We are pleased that many genes in this specific set have now been found, but again conclude that their presence or absence in chicken has little bearing on the question of whether or not missing avian genes have been lost in syntenic blocks.

Lovell et al. [1] did not claim that their avian missing gene list was complete, or error-free. In fact, our analysis of the new PacBio-based chicken genome (Galgal5; [3]) and/or other avian genomes more recently available in NCBI has revealed the presence of seven of the eight genes reported by Bornelöv et al. [4], the majority of those previously reported by Hron et al. [2], and an additional small subset of the 274 genes originally reported as missing in all birds. Nevertheless, 232 out of the original 274 genes (approximately 85%) reported missing in all birds still remain missing. Warren et al. [3] have also found in Galgal5 another set of 240 genes previously not found in the chicken genome but present in other birds, including 29 out of the 39 genes previously found only in other birds and now being reported by Bornelöv et al. in chicken. It is reassuring that Bornelöv et al. have found RNAseq evidence for those genes, and were able to map several of them to the corresponding loci in Galgal5. Overall, these observations support the presence and expression of several genes previously thought to be missing in birds, and thus are certainly important for a better understanding of avian biology. However, they include only a very limited set of the genes reported as missing in all birds by Lovell et al. [1]; the majority of those genes have not yet been found in any bird species, in spite of very extensive searches of various genomes and transcriptome databases.

In their conclusion, Bornelöv et al. [4] make a further claim that 80% of their newly found genes in chicken (including an undisclosed number that brings the total to 191) map to microchromosomes, and that these genes are also present in syntenic clusters. A careful examination, however, shows that more than 80% of these genes remain unplaced in Galgal5; moreover, whereas approximately 21% of these genes occur as groups in the same contigs, 78% are on a unique contig. Little or no synteny information is available, so it is not possible to draw accurate conclusions about a possible organization of newly found genes in clusters. It is also not possible to establish orthology conclusively for most of these genes so as to exclude possible lineage-specific paralogs (examples in Table S3 of Lovell et al. [1] and in Table S3 of Warren et al. [3]). Furthermore, recent improvements in assembly quality continue to provide increasing evidence that genes that flank the avian missing syntenic blocks are present and adjacent to each other in birds, supporting the notion that some missing blocks may have been lost as a result of chromosomal rearrangements, as suggested in Lovell et al. [1]. Examples are in Table S4 from Warren et al. [3], or can be seen by examining the relevant syntenic regions in the current avian genome assemblies in NCBI.

Bornelöv et al. [4] state at the end of their Abstract: ‘Hence, the occurrence of syntenic groups of vertebrate genes that have not been observed in Aves does not prove the evolutionary loss of such genes’. Lovell et al. [1] did indeed provide evidence that a large proportion of avian missing genes are present in syntenic clusters that are conserved in non-avian organisms. However, we did not conclude that the simple occurrence of those missing genes in syntenic clusters was of itself proof of their evolutionary loss in birds. In fact, we employed extensive curatorial and comparative efforts for each missing gene, and were conservative in removing any genes for which suggestive evidence hinted at their presence in birds. The fact that a large number of avian missing genes are part of conserved syntenic blocks in non-avian organisms remains largely unchallenged. Interestingly, we have noted that genes that were previously found only in one or a few avian species, and now are found on microchromosomes or on difficult to assemble unplaced segments (as described in Table S6 in Lovell et al. [1]; by Hron et al. [2]; by Warren et al. [3]; and in Table 1 in Bornelöv et al. [4]), tend to share low sequence identity (often less than 40%) with orthologs in other sauropsids (lizards, crocodiles, or turtles) and in mammals. Thus, these genes appear to have diverged significantly in the avian lineage, or appear to be truncated or partial, and thus may represent pseudogenes. A better evaluation of their completeness and conservation, together with mechanistic studies, will be crucial in revealing how these genes function in the context of key pathways and networks that influence avian physiology.

## Conclusions

In summary, we find the evidence presented in Bornelöv et al. [4] informative, but it does not significantly challenge the main findings and conclusions in Lovell et al. [1]. To the contrary, by showing that a core set of otherwise conserved vertebrate protein-coding genes cannot be found in chicken in spite of exhaustive searches of a large set of transcriptome databases, this study actually provides supportive evidence that these genes do indeed represent avian missing genes. We believe that a systematic application of the newest available methodologies (for example, PacBio sequencing), coupled with extended and more comprehensive transcriptome analyses, will improve the genome representation of difficult to read and/or assemble regions and will contribute to clarifying the exact extent of avian gene losses.

## Abbreviations

NCBI: National Center for Biotechnology Information
SRA: Sequence Read Archive
